# Bis(melaminium) succinate succinic acid monosolvate dihydrate

**DOI:** 10.1107/S1600536812033387

**Published:** 2012-07-25

**Authors:** Barbara Froschauer, Matthias Weil

**Affiliations:** a Institute for Applied Synthetic Chemistry, Vienna University of Technology, Getreidemarkt 9/163, A-1060 Vienna, Austria; b Institute for Chemical Technologies and Analytics, Division of Structural Chemistry, Vienna University of Technology, Getreidemarkt 9/164-SC, A-1060 Vienna, Austria

## Abstract

The asymmetric unit of the solvated title salt, 2C_3_H_7_N_6_^+^·C_4_H_4_O_4_^2−^·C_4_H_6_O_4_·2H_2_O, contains one essentially planar melaminium (2,4,6-triamino-1,3,5-triazin-1-ium) cation (r.m.s. deviation of the non-H atoms = 0.0097 Å), one-half of a succinate anion, one-half of a succinic acid solvent mol­ecule and one water molecule of crystallization; full mol­ecules are generated by inversion symmetry. Supra­molecular layers parallel to (12-1) are formed through extensive inter­molecular hydrogen bonding of the types O—H⋯O, N—H⋯N and N—H⋯O between the components.

## Related literature

For the use of melaminium salts in polymer science, see: Weinstabl *et al.* (2001 ▶). For a list of structurally determined melaminium salts of purely organic carb­oxy­lic acids, see: Froschauer & Weil (2012 ▶).
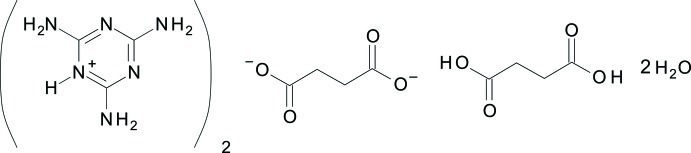


## Experimental

### 

#### Crystal data

2C_3_H_7_N_6_^+^·C_4_H_4_O_4_^2−^·C_4_H_6_O_4_·2H_2_O
*M* *_r_* = 524.48
Triclinic, 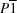

*a* = 7.1193 (7) Å
*b* = 8.1650 (8) Å
*c* = 9.5595 (9) Å
α = 88.013 (2)°
β = 84.647 (2)°
γ = 88.093 (2)°
*V* = 552.68 (9) Å^3^
*Z* = 1
Mo *K*α radiation
μ = 0.13 mm^−1^
*T* = 293 K
0.23 × 0.18 × 0.12 mm

#### Data collection

Siemens SMART CCD diffractometer
5533 measured reflections
2719 independent reflections
1545 reflections with *I* > 2σ(*I*)
*R*_int_ = 0.028

#### Refinement

*R*[*F* ^2^ > 2σ(*F* ^2^)] = 0.047
*wR*(*F* ^2^) = 0.142
*S* = 0.97
2719 reflections
173 parameters
3 restraints
H atoms treated by a mixture of independent and constrained refinement
Δρ_max_ = 0.30 e Å^−3^
Δρ_min_ = −0.26 e Å^−3^

### 

Data collection: *SMART* (Siemens, 1996 ▶); cell refinement: *SAINT* (Siemens, 1996 ▶); data reduction: *SAINT*; program(s) used to solve structure: *SHELXS97* (Sheldrick, 2008 ▶); program(s) used to refine structure: *SHELXL97* (Sheldrick, 2008 ▶); molecular graphics: *Mercury* (Macrae *et al.*, 2006 ▶) and *ATOMS* (Dowty, 2006 ▶); software used to prepare material for publication: *publCIF* (Westrip, 2010 ▶).

## Supplementary Material

Crystal structure: contains datablock(s) I, global. DOI: 10.1107/S1600536812033387/cv5324sup1.cif

Structure factors: contains datablock(s) I. DOI: 10.1107/S1600536812033387/cv5324Isup2.hkl

Supplementary material file. DOI: 10.1107/S1600536812033387/cv5324Isup3.cml

Additional supplementary materials:  crystallographic information; 3D view; checkCIF report

## Figures and Tables

**Table 1:** Hydrogen-bond geometry (Å, °)

*D*—H⋯*A*	*D*—H	H⋯*A*	*D*⋯*A*	*D*—H⋯*A*
N4—H3⋯N2^i^	0.86	2.10	2.959 (2)	176
N1—H1⋯O1	0.86	2.01	2.844 (2)	164
N5—H4⋯O3^ii^	0.86	2.13	2.976 (2)	170
N6—H6⋯N3^iii^	0.86	2.16	3.015 (2)	173
N1—H1⋯O2	0.86	2.50	3.199 (2)	138
N4—H2⋯O3^iv^	0.86	2.14	2.799 (2)	134
N4—H2⋯O1	0.86	2.56	3.268 (2)	141
N6—H7⋯O2	0.86	1.94	2.782 (2)	166
N5—H5⋯O1*W* ^v^	0.86	2.11	2.912 (2)	154
O1*W*—H1*W*⋯O4	0.88 (2)	2.35 (2)	3.195 (2)	162 (3)
O1*W*—H2*W*⋯O2^vi^	0.86 (2)	1.89 (2)	2.726 (2)	167 (3)
O4—H12⋯O1^iv^	1.02 (2)	1.55 (2)	2.5673 (19)	177 (3)

Symmetry codes: (i) 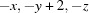; (ii) 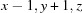; (iii) 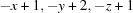; (iv) 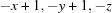; (v) 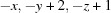; (vi) 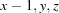.

**Fig. 1.**
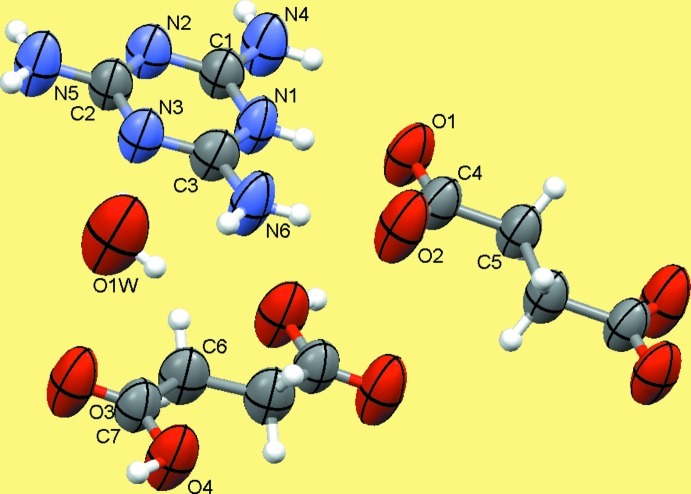
The molecular components of the title compound drawn with atomic displacement factors at the 90% probability level. H atoms are displayed as spheres with an arbirtary radius. For the centrosymmetric succinate anion and the succinic acid solvent molecule the symmetry-equivalent atoms are not labelled.

**Fig. 2.**
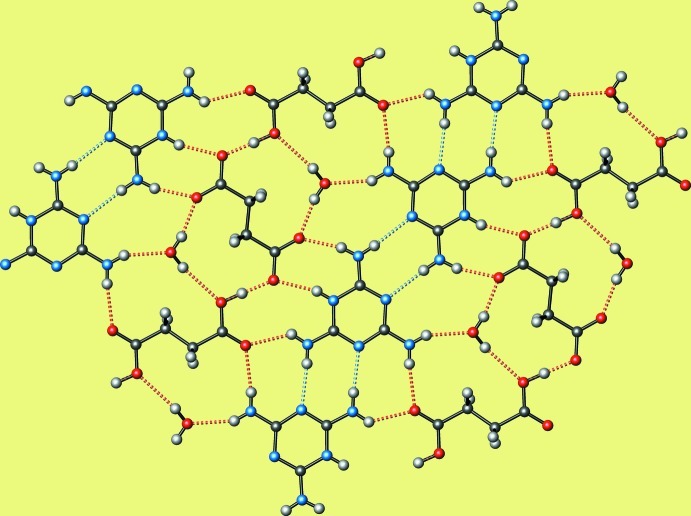
Supramolecular layer built up through hydrogen bonding interactions (dashed lines) between the molecular components.

**Fig. 3.**
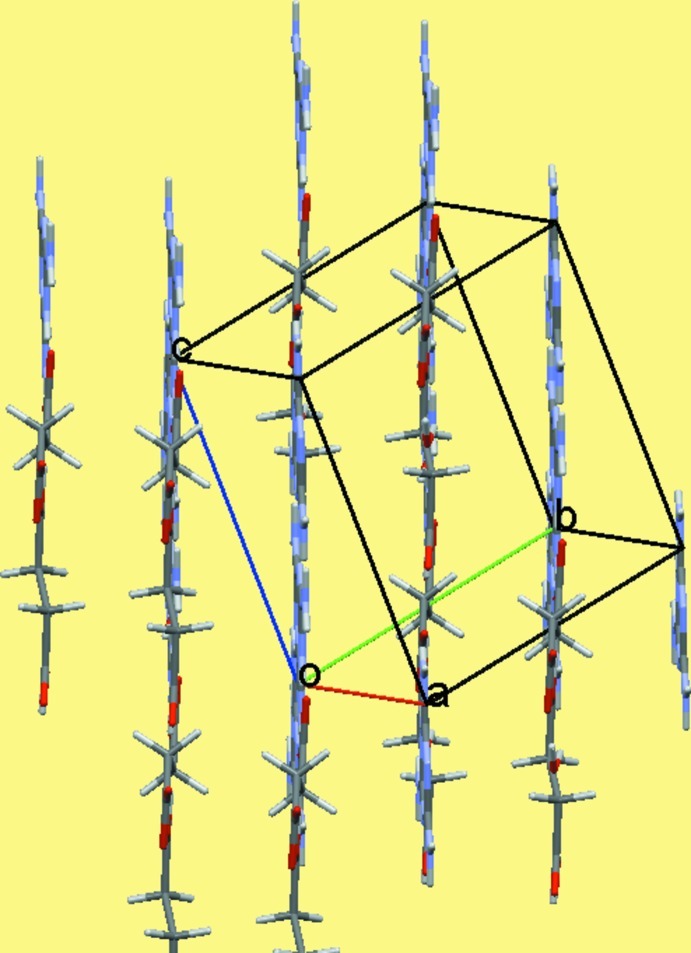
The assembly of supramolecular layers in the crystal parallel to (121).

## supplementary crystallographic information

### Comment

The potential substitution of melamine through organic melaminium salts for production of melamine urea formaldehyde (MUF) resins (Weinstabl *et al.*, 2001) render the structural investigation of these compounds interesting. A list of already determined structures of purely organic melaminium salts has been compiled recently by Froschauer & Weil (2012).

The *pK_a_* values of 4.21 and 5.72 for the first and second deprotonation step of succinic acid (the *pK_a_* of the first deprotonation step of melamine is 5.10) led to a doubly deprotonated anion in the title compound, bis-melaminium succinate succinic acid solvate dihydrate, 2(C_3_H_7_N_6_)^+.^C_4_H_4_O_4_^-.^C_4_H_6_O_4_^.^2(H_2_O). However, besides lattice water, there is also one succinic acid solvent molecule present in the unit cell. The succinic acid molecule and the succinate anion are located with their central C—C bond on inversion centres. As observed for all single protonated melaminium cations, the protonation of melamine takes place at one of the triazine N ring atoms (Fig. 1).

The melaminium cation is essentially planar with a r.m.s. deviation of 0.0097 Å. Likewise, the anion (r.m.s. deviation 0.039 Å) and the succinic acid molecule (r.m.s. deviation 0.060 Å) can be considered as planar. The angles between the least-squares planes of 6.59 (9) ° and 5.76 (12) ° between the anion and the cation and the succinic acid molecule, respectively, lead to the formation of supramolecular layers where cations are arranged in rows alternating with rows of anions, succinic acid solvent and lattice water molecules (Fig. 2). Extensive intermolecular hydrogen bonding of the types O—H···O, N—H···N and N—H···O between the molecular components is present. Details are reported in Table 1. The motif for the hydrogen-bonded assembly of two melaminium cations in such a layer is the same as in the hydrogenmalonate salt and other melaminium salts (Froschauer & Weil, 2012). In the crystal, the supramolecular layers are arranged parallel to (121) (Fig. 3) with an interplanar distance of approximately 3.15 Å.

### Experimental

79.3 mmol melamine was dissolved under refluxing conditions in 200 ml distilled water. The stoichiometric quantity (1:1) of succinic acid was added within five minutes. The mixture was then refluxed for 30 minutes and then cooled to room temperature. The precipitate formed on cooling was separeted by filtration and washed with cold methanol. The crystalline product was then dried *in vacuo* at 303–313 K. Single crystal growth was accomplished by dissolution of 1 g of the crystalline product under refluxing conditions in an aqueous methanol solution (2:1 *v*/*v*) to get a saturated solution. Then the solution was slowly cooled down to room temperature. Suitable crystals were obtained by slow evaporation of the solvents during five days. The crystals were washed with methanol and dried *in vacuo* at room temperature giving analytical pure samples. CHN analysis (found/calc.): C (32.13/32.10), H (5.50/5.38), N (31.93/32.05). NMR: (solution, DMSO) chemical shift [p.p.m.]: ^1^H 10.37 (s, 2H), 6.22 (s, 6H), 2.39 (s, 4H); ^13^C 174.32, 166.43, 29.29.

### Refinement

The proton at the triazine ring of the melaminium cation was clearly discernible from a difference Fourier map (like all other H atoms). For refinement, the H atoms attached to C or N atoms were set in calculated positions and treated as riding on their parent atoms with C—H = 0.97 Å and N—H = 0.86 Å and with *U*_iso_(H) = 1.2*U*_eq_(C,*N*). The proton of the carboxy group of the succinic acid solvent molecule was refined with a distance restraint O—H = 1.00 (2) Å; H atoms of the water molecule were likewise refined with a distance restraint of O—H = 0.88 (2) Å.

### Crystal data

**Table:**

2C_3_H_7_N_6_^+^·C_4_H_4_O_4_^2^^−^·C_4_H_6_O_4_·2H_2_O	*Z* = 1
*M**_r_* = 524.48	*F*(000) = 276
Triclinic, *P*1	*D*_x_ = 1.576 Mg m^−^^3^
Hall symbol: -P 1	Mo *K*α radiation, λ = 0.71073 Å
*a* = 7.1193 (7) Å	Cell parameters from 1570 reflections
*b* = 8.1650 (8) Å	θ = 2.5–28.8°
*c* = 9.5595 (9) Å	µ = 0.13 mm^−^^1^
α = 88.013 (2)°	*T* = 293 K
β = 84.647 (2)°	Parallelepiped, colourless
γ = 88.093 (2)°	0.23 × 0.18 × 0.12 mm
*V* = 552.68 (9) Å^3^	

### Data collection

**Table:**

Siemens SMART CCD diffractometer	1545 reflections with *I* > 2σ(*I*)
Radiation source: fine-focus sealed tube	*R*_int_ = 0.028
Graphite monochromator	θ_max_ = 28.3°, θ_min_ = 2.1°
ω scans	*h* = −9→9
5533 measured reflections	*k* = −10→10
2719 independent reflections	*l* = −12→12

### Refinement

**Table:**

Refinement on *F*^2^	Secondary atom site location: difference Fourier map
Least-squares matrix: full	Hydrogen site location: inferred from neighbouring sites
*R*[*F*^2^ > 2σ(*F*^2^)] = 0.047	H atoms treated by a mixture of independent and constrained refinement
*wR*(*F*^2^) = 0.142	*w* = 1/[σ^2^(*F*_o_^2^) + (0.0791*P*)^2^] where *P* = (*F*_o_^2^ + 2*F*_c_^2^)/3
*S* = 0.97	(Δ/σ)_max_ < 0.001
2719 reflections	Δρ_max_ = 0.30 e Å^−^^3^
173 parameters	Δρ_min_ = −0.26 e Å^−^^3^
3 restraints	Extinction correction: *SHELXL97* (Sheldrick, 2008), Fc^*^=kFc[1+0.001xFc^2^λ^3^/sin(2θ)]^-1/4^
Primary atom site location: structure-invariant direct methods	Extinction coefficient: 0.010 (4)

### Special details

**Table:**

Geometry. All e.s.d.'s (except the e.s.d. in the dihedral angle between two l.s. planes) are estimated using the full covariance matrix. The cell e.s.d.'s are taken into account individually in the estimation of e.s.d.'s in distances, angles and torsion angles; correlations between e.s.d.'s in cell parameters are only used when they are defined by crystal symmetry. An approximate (isotropic) treatment of cell e.s.d.'s is used for estimating e.s.d.'s involving l.s. planes.
Refinement. Refinement of *F*^2^ against ALL reflections. The weighted *R*-factor *wR* and goodness of fit *S* are based on *F*^2^, conventional *R*-factors *R* are based on *F*, with *F* set to zero for negative *F*^2^. The threshold expression of *F*^2^ > σ(*F*^2^) is used only for calculating *R*-factors(gt) *etc*. and is not relevant to the choice of reflections for refinement. *R*-factors based on *F*^2^ are statistically about twice as large as those based on *F*, and *R*- factors based on ALL data will be even larger.

### Fractional atomic coordinates and isotropic or equivalent isotropic displacement parameters (Å^2^)

**Table:**

	*x*	*y*	*z*	*U*_iso_*/*U*_eq_	
N3	0.3081 (2)	1.0330 (2)	0.36672 (15)	0.0308 (4)	
N1	0.4064 (2)	0.8895 (2)	0.16163 (15)	0.0323 (4)	
H1	0.4894	0.8279	0.1160	0.039*	
O1	0.62741 (19)	0.68205 (18)	−0.02657 (14)	0.0385 (4)	
O3	0.6682 (2)	0.2996 (2)	0.25900 (15)	0.0456 (4)	
O2	0.8071 (2)	0.6984 (2)	0.14806 (15)	0.0480 (5)	
O4	0.3997 (2)	0.44613 (19)	0.26505 (14)	0.0431 (4)	
C7	0.5535 (3)	0.3930 (3)	0.3191 (2)	0.0309 (5)	
C1	0.2450 (3)	0.9365 (2)	0.10469 (19)	0.0284 (4)	
N2	0.1121 (2)	1.0287 (2)	0.17327 (15)	0.0305 (4)	
C2	0.1488 (3)	1.0729 (2)	0.30373 (18)	0.0281 (4)	
C6	0.5839 (3)	0.4567 (3)	0.46117 (19)	0.0339 (5)	
H6A	0.6871	0.5321	0.4491	0.041*	
H6B	0.6233	0.3651	0.5200	0.041*	
N4	0.2251 (2)	0.8874 (2)	−0.02317 (16)	0.0370 (4)	
H3	0.1250	0.9151	−0.0632	0.044*	
H2	0.3123	0.8277	−0.0664	0.044*	
N6	0.5975 (2)	0.8950 (2)	0.34112 (18)	0.0402 (5)	
H6	0.6226	0.9242	0.4228	0.048*	
H7	0.6778	0.8356	0.2909	0.048*	
C3	0.4365 (3)	0.9412 (2)	0.29314 (19)	0.0301 (5)	
C4	0.7771 (3)	0.6435 (2)	0.0324 (2)	0.0308 (5)	
N5	0.0177 (2)	1.1639 (2)	0.37353 (17)	0.0396 (5)	
H5	0.0347	1.1959	0.4561	0.048*	
H4	−0.0848	1.1912	0.3365	0.048*	
O1W	0.0343 (3)	0.6661 (3)	0.36216 (18)	0.0637 (6)	
H1W	0.126 (4)	0.591 (3)	0.353 (3)	0.097*	
H2W	−0.024 (4)	0.667 (4)	0.288 (2)	0.097*	
H12	0.385 (4)	0.393 (3)	0.171 (2)	0.097*	
C5	0.9192 (3)	0.5292 (3)	−0.04228 (19)	0.0306 (5)	
H5A	0.9717	0.5841	−0.1280	0.037*	
H5B	0.8543	0.4341	−0.0690	0.037*	

### Atomic displacement parameters (Å^2^)

**Table:**

	*U*^11^	*U*^22^	*U*^33^	*U*^12^	*U*^13^	*U*^23^
N3	0.0277 (9)	0.0441 (10)	0.0218 (8)	0.0109 (8)	−0.0092 (7)	−0.0101 (7)
N1	0.0283 (9)	0.0444 (10)	0.0248 (8)	0.0147 (8)	−0.0070 (7)	−0.0131 (7)
O1	0.0295 (8)	0.0537 (10)	0.0344 (8)	0.0173 (7)	−0.0150 (6)	−0.0151 (7)
O3	0.0392 (9)	0.0659 (11)	0.0337 (8)	0.0230 (8)	−0.0151 (7)	−0.0236 (7)
O2	0.0455 (9)	0.0664 (11)	0.0351 (8)	0.0258 (8)	−0.0193 (7)	−0.0285 (8)
O4	0.0381 (9)	0.0635 (11)	0.0303 (8)	0.0209 (7)	−0.0178 (6)	−0.0191 (7)
C7	0.0278 (10)	0.0395 (12)	0.0261 (9)	0.0081 (9)	−0.0067 (8)	−0.0069 (8)
C1	0.0269 (10)	0.0366 (11)	0.0222 (9)	0.0051 (8)	−0.0061 (8)	−0.0061 (8)
N2	0.0281 (9)	0.0428 (10)	0.0217 (8)	0.0076 (7)	−0.0085 (7)	−0.0097 (7)
C2	0.0250 (10)	0.0386 (11)	0.0215 (9)	0.0063 (8)	−0.0062 (8)	−0.0067 (8)
C6	0.0296 (11)	0.0479 (13)	0.0257 (10)	0.0076 (9)	−0.0106 (8)	−0.0085 (9)
N4	0.0332 (9)	0.0548 (12)	0.0245 (8)	0.0131 (8)	−0.0107 (7)	−0.0169 (8)
N6	0.0330 (10)	0.0596 (12)	0.0295 (9)	0.0231 (9)	−0.0130 (7)	−0.0173 (8)
C3	0.0301 (10)	0.0372 (12)	0.0243 (9)	0.0055 (9)	−0.0095 (8)	−0.0070 (8)
C4	0.0282 (10)	0.0357 (11)	0.0294 (10)	0.0105 (9)	−0.0083 (8)	−0.0083 (8)
N5	0.0322 (9)	0.0630 (13)	0.0255 (8)	0.0174 (9)	−0.0127 (7)	−0.0182 (8)
O1W	0.0646 (12)	0.0902 (15)	0.0408 (9)	0.0294 (10)	−0.0287 (8)	−0.0315 (9)
C5	0.0299 (10)	0.0388 (12)	0.0243 (9)	0.0108 (9)	−0.0087 (8)	−0.0104 (8)

### Geometric parameters (Å, º)

**Table:**

N3—C3	1.328 (2)	C6—H6A	0.9700
N3—C2	1.358 (2)	C6—H6B	0.9700
N1—C1	1.356 (2)	N4—H3	0.8600
N1—C3	1.378 (2)	N4—H2	0.8600
N1—H1	0.8600	N6—C3	1.313 (2)
O1—C4	1.277 (2)	N6—H6	0.8600
O3—C7	1.217 (2)	N6—H7	0.8600
O2—C4	1.246 (2)	C4—C5	1.500 (3)
O4—C7	1.309 (2)	N5—H5	0.8600
O4—H12	1.023 (17)	N5—H4	0.8600
C7—C6	1.507 (3)	O1W—H1W	0.882 (17)
C1—N4	1.321 (2)	O1W—H2W	0.856 (18)
C1—N2	1.328 (2)	C5—C5^ii^	1.522 (3)
N2—C2	1.361 (2)	C5—H5A	0.9700
C2—N5	1.319 (2)	C5—H5B	0.9700
C6—C6^i^	1.514 (4)		
			
C3—N3—C2	115.93 (15)	C1—N4—H2	120.0
C1—N1—C3	119.48 (16)	H3—N4—H2	120.0
C1—N1—H1	120.3	C3—N6—H6	120.0
C3—N1—H1	120.3	C3—N6—H7	120.0
C7—O4—H12	111.6 (17)	H6—N6—H7	120.0
O3—C7—O4	122.87 (17)	N6—C3—N3	122.22 (17)
O3—C7—C6	121.05 (18)	N6—C3—N1	116.61 (17)
O4—C7—C6	116.07 (17)	N3—C3—N1	121.17 (17)
N4—C1—N2	120.96 (17)	O2—C4—O1	121.88 (17)
N4—C1—N1	117.14 (17)	O2—C4—C5	120.22 (17)
N2—C1—N1	121.90 (16)	O1—C4—C5	117.90 (16)
C1—N2—C2	115.70 (16)	C2—N5—H5	120.0
N5—C2—N3	117.76 (16)	C2—N5—H4	120.0
N5—C2—N2	116.44 (16)	H5—N5—H4	120.0
N3—C2—N2	125.80 (17)	H1W—O1W—H2W	107 (3)
C7—C6—C6^i^	116.3 (2)	C4—C5—C5^ii^	115.00 (19)
C7—C6—H6A	108.2	C4—C5—H5A	108.5
C6^i^—C6—H6A	108.2	C5^ii^—C5—H5A	108.5
C7—C6—H6B	108.2	C4—C5—H5B	108.5
C6^i^—C6—H6B	108.2	C5^ii^—C5—H5B	108.5
H6A—C6—H6B	107.4	H5A—C5—H5B	107.5
C1—N4—H3	120.0		

Symmetry codes: (i) −*x*+1, −*y*+1, −*z*+1; (ii) −*x*+2, −*y*+1, −*z*.

### Hydrogen-bond geometry (Å, º)

**Table:**

*D*—H···*A*	*D*—H	H···*A*	*D*···*A*	*D*—H···*A*
N4—H3···N2^iii^	0.86	2.10	2.959 (2)	176
N1—H1···O1	0.86	2.01	2.844 (2)	164
N5—H4···O3^iv^	0.86	2.13	2.976 (2)	170
N6—H6···N3^v^	0.86	2.16	3.015 (2)	173
N1—H1···O2	0.86	2.50	3.199 (2)	138
N4—H2···O3^vi^	0.86	2.14	2.799 (2)	134
N4—H2···O1	0.86	2.56	3.268 (2)	141
N6—H7···O2	0.86	1.94	2.782 (2)	166
N5—H5···O1*W*^vii^	0.86	2.11	2.912 (2)	154
O1*W*—H1*W*···O4	0.88 (2)	2.35 (2)	3.195 (2)	162 (3)
O1*W*—H2*W*···O2^viii^	0.86 (2)	1.89 (2)	2.726 (2)	167 (3)
O4—H12···O1^vi^	1.02 (2)	1.55 (2)	2.5673 (19)	177 (3)

Symmetry codes: (iii) −*x*, −*y*+2, −*z*; (iv) *x*−1, *y*+1, *z*; (v) −*x*+1, −*y*+2, −*z*+1; (vi) −*x*+1, −*y*+1, −*z*; (vii) −*x*, −*y*+2, −*z*+1; (viii) *x*−1, *y*, *z*.
